# Engineering a Second Interchain Disulfide Bond in the αβ T-Cell Receptor Constant Domain: A Powerful Strategy to Enhance Stability, Pairing Fidelity, and Therapeutic Efficacy in TCR-T Cell Therapy

**DOI:** 10.3390/ph19060888

**Published:** 2026-06-03

**Authors:** Nguyen Trung Quan, Xiangliang Lin, Duong Thi Nhu Xuan, Bui Thi Van Anh

**Affiliations:** 1 Department of Biomedical Sciences, City University of Hong Kong, Kowloon, Hong Kong 999077, China; nguyentrungquan6991@gmail.com; 2 Esco Aster Pte. Ltd., Singapore 139950, Singapore; xl.lin@escoaster.com; 3 College of Medical Science and Technology, Taipei Medical University, Taipei 11031, Taiwan; duongthinhuxuan1002@gmail.com; 4 Faculty of Pharmacy, Ton Duc Thang University, Ho Chi Minh City 700000, Vietnam

**Keywords:** disulfide bond, constant domain, immunotherapy, TCR-T

## Abstract

Adoptive TCR-T cell therapy holds great promise for personalised cancer treatment, yet it is limited by poor surface expression, chain mispairing, and suboptimal stability of introduced TCRs. One effective structure-guided approach is to introduce a second interchain disulfide bond between the α and β constant domains. This review summarises the structural and mechanistic basis of this strategy, its impact on TCR folding, CD3 assembly, mechanotransduction, and anti-tumour function, as well as current engineering approaches, persistent challenges, and future perspectives. Preclinical studies demonstrate improved heterodimer stability, reduced mispairing, higher surface expression, and enhanced signalling, positioning the second disulfide bond as a valuable complementary tool in next-generation TCR engineering.

## 1. Introduction of TCR Structure and Functions

The T cell receptor (TCR) is a cell surface receptor expressed on T lymphocytes that mediates antigen recognition and plays a central role in cell-mediated immune responses [1]. TCRs are classified into two major groups: αβTCRs, which account for more than 90% of all TCRs, and γδTCRs, which represent less than 10% in humans [1]. Among these, αβTCRs are primarily responsible for recognising peptide antigens presented by major histocompatibility complex (MHC) molecules and constitute the most widely exploited TCR type in current T-cell-based immunotherapies, particularly TCR-T cell therapy [2]. Structurally, αβTCRs exist as heterodimers composed of two non-identical polypeptide chains, the α (alpha) and β (beta) chains, which are linked by a disulfide bond [3]. Each TCR chain consists of a variable domain and a constant domain, as well as a hinge region, a transmembrane region, and a short cytoplasmic tail, all of which collectively contribute to receptor stability and intracellular signal transduction [4].

Variable domains, including α (αV) and β (βV), belong to the immunoglobulin superfamily [5]. They share the same structure as the variable region of antibodies [5]. This region plays a main role in the interaction between peptide-MHC presented on antigen-presenting cells (APC) and the TCR on the T cell surface [2]. This domain comprises the three core regions with complementary abilities (complementarity-determining regions—CDRs), which are designated as CDR1, CDR2, and CDR3 [6]. CDR1 and CDR2 are encoded by the gene V and are responsible for interacting with the α-helix chain in MHC structures [3,6]. On the other hand, CDR3 is the most variable region, which is a fusion of gene V, gene D, and gene J [7]. CDR3 is the key to TCR function due to its specific recognition and binding to antigenic peptides [7]. The diversity of the αβTCR is the result of the complicated adaptive immune system–specific gene recombination mechanism [2]. V(D)J recombination involves the random combination of V, D (present only in the β chain), and J gene segments, together with template-independent nucleotide addition or deletion (N/P nucleotide addition) at the junctions [8]. These mechanisms generate an extraordinarily diverse TCR repertoire, enabling the T-cell immune system to recognise an almost unlimited array of distinct peptide epitopes [8]. Consequently, the variable domain directly determines the antigen specificity of each individual TCR.

In contrast to the variable domains, the constant domains (αC and βC) exhibit a much lower degree of diversity and primarily serve structural roles [3]. These domains contribute to maintaining the stable three-dimensional conformation of the TCR, ensuring the proper assembly of the αβTCR heterodimer and its association with accompanying signalling molecules [3]. One of the key functions of the constant domains is their interaction with the CD3 complex (γεCD3, δεCD3, and ζζCD3), which is responsible for transmitting activation signals into the T cell, as the TCR itself lacks intracellular signalling motifs [9]. Through these interactions, the constant domains play an indirect yet essential role in the initiation of TCR signalling [9]. Notably, the βC domain has been shown to be particularly important in TCR physiology [3]. This domain contains a distinctive structural element known as the FG loop, which connects the β-strand F to the G and is capable of participating in mechanotransduction [10]. When the TCR engages peptide–MHC (pMHC) under mechanical forces generated during contact between T cells and APCs, the FG loop of βC is thought to contribute to the conversion of mechanical signals into biochemical signals, thereby enhancing the efficiency of T cell activation [1,10].

## 2. Functional Specialisation-Based Design of the TCR System

Although the αβTCR can recognise pMHC complexes with high specificity, the TCR itself is unable to transmit intracellular signals [2]. T cell activation can only occur when the TCR functions in close coordination with the CD3 complex, a group of accessory molecules that play an essential role in signal transduction [2,9]. Structurally, the cytoplasmic tails of both the αTCR and βTCR chains are extremely short, consisting of only a few amino acids [9]. Notably, these tails lack immunoreceptor tyrosine-based activation motifs (ITAMs), which are critical for initiating tyrosine kinase-dependent signalling cascades in immune cells [11]. Owing to the absence of these intracellular signalling motifs, the αβTCR complex is restricted to antigen recognition and cannot directly trigger intracellular signalling pathways [11]. This architectural feature establishes a clear functional division within the TCR complex, with the TCR mediating antigen recognition and CD3 responsible for signal transduction [11].

**Figure 1:**
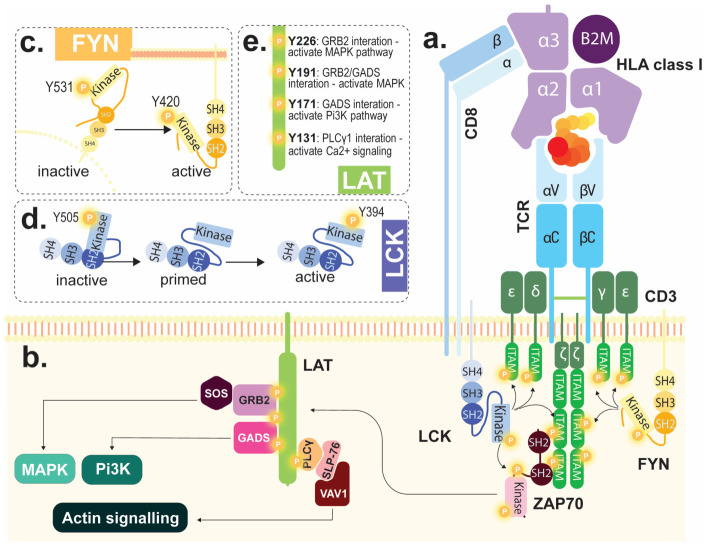
TCR-mediated signal transduction. (**a**) Upon recognition of peptide–MHC complexes by the T-cell receptor (TCR), conformational changes and clustering of the TCR–CD3 complex trigger phosphorylation of immunoreceptor tyrosine-based activation motifs (ITAMs) in the CD3 chains. (**b**) Phosphorylated-CD3 recruits and activates LCK, FYN, and ZAP-70, which in turn phosphorylate downstream adaptors and signalling molecules, ultimately leading to T-cell activation, proliferation, and effector function. (**c**) FYN is activated upon CD45-mediated dephosphorylation of Tyr531, thereby releasing SH2 inhibition; subsequent phosphorylation of Tyr420 enables downstream substrate phosphorylation. (**d**) LCK is activated by CD45-mediated dephosphorylation of Tyr505, releasing SH2 autoinhibition; subsequent Tyr394 phosphorylation enables downstream TCR signalling. (**e**) LAT is phosphorylated by ZAP-70 on key tyrosines (Tyr131, Tyr171, Tyr191, and Tyr226) upon TCR activation, serving as a scaffold to recruit signalling proteins and propagate downstream TCR signalling.

On the T cell membrane, the TCR exists as a fully assembled multimeric complex, in which the αβTCR heterodimer is non-covalently associated with the CD3 chains (Figure 1) [3,9]. The CD3 complex comprises three main components: the γεCD3 heterodimer, the δεCD3 heterodimer, and the ζζCD3 homodimer [9]. Each CD3 chain contains between one and three ITAMs, with CD3ζ displaying the highest ITAM density (three ITAMs per chain) [9,11]. In total, a complete TCR–CD3 complex harbours approximately ten ITAMs, providing the molecular basis for signal amplification following antigen recognition by the TCR [11]. Beyond their signalling function, the CD3 chains also contribute to stabilising TCR surface expression, as the αβTCR heterodimer cannot be efficiently transported to the plasma membrane in the absence of the associated CD3 complex [11].

T cell signal transduction is initiated when the TCR recognises and binds to pMHC complexes presented on APCs [12]. This interaction is not purely chemical in nature but also involves mechanical forces generated during the physical contact between T cells and APCs [12]. These mechanical forces induce conformational changes within the TCR–CD3 complex, leading to the exposure of ITAMs located in the cytoplasmic tails of CD3 (Figure 1) [11]. The exposed ITAMs are subsequently phosphorylated by Src-family kinases, predominantly Lck, which is positioned in close proximity to the plasma membrane through association with the CD4 or CD8 coreceptors [11,13]. Once phosphorylated, the ITAMs serve as docking sites for ZAP-70, a central kinase in TCR signalling [14]. Activated ZAP-70 phosphorylates key adaptor proteins, particularly LAT (linker for activation of T cells), thereby promoting the assembly of multiprotein signalling complexes [14]. Activation of the LAT axis subsequently triggers multiple downstream signalling pathways, including the MAPK cascade, as well as key transcription factors such as NFAT, NF-κB, and AP-1 [15]. Together, these transcriptional regulators coordinate the expression of genes involved in T cell proliferation, differentiation, and effector functions [11].

## 3. Factors Affecting the Stability of TCR Cell-Surface Presentation

The sustained presence of the TCR on the surface of T cells is not the result of a passive process but is instead tightly regulated by multiple layers of quality control mechanisms during biosynthesis, protein folding, and intracellular trafficking. Only fully assembled TCR–CD3 complexes that meet stringent structural requirements are able to escape the quality control system of the endoplasmic reticulum (ER) and be stably expressed on the plasma membrane [9]. The αTCR chain, βTCR chain, and the CD3 subunits are glycoproteins that undergo N-linked glycosylation during co-translational and post-translational processing in the ER and the Golgi apparatus [16]. The glycosylation process plays a critical role in promoting proper protein folding and facilitating the correct assembly of the TCR–CD3 complex [17]. In addition to its structural function, glycosylation helps TCR and CD3 chains evade recognition as misfolded proteins by the ER quality control machinery, thereby preventing endoplasmic reticulum-associated degradation (ERAD) [18,19]. Through this mechanism, glycosylation indirectly regulates the surface density of TCRs on T cells, influencing the activation threshold and the strength of TCR signalling [20]. Conversely, defects in glycosylation—caused either by mutations at glycosylation sites or by dysfunction of glycosyltransferase enzymes—can result in ER retention of the TCR, activation of ERAD pathways, and a marked reduction in TCR surface expression [19].

The αβTCR heterodimer cannot exist independently on the T cell surface in the absence of association with the CD3 chains [9]. Assembly of the TCR–CD3 complex is governed by specific ionic interactions within the transmembrane regions [9]. Specifically, the αTCR and βTCR chains contain positively charged amino acid residues, whereas the CD3 chains harbour negatively charged residues [9]. This electrostatic complementarity enables the formation of stable ionic interactions, ensuring proper assembly of the complete TCR–CD3 complex and permitting its transport to the cell surface [21]. When the TCR fails to associate with CD3, the TCR chains are recognised as incomplete or misassembled structures, leading to intracellular degradation and complete absence of surface expression [21]. Thus, CD3 functions not only as the signalling apparatus of the TCR but also as a critical determinant of the physical existence of the TCR on the T cell surface [21].

The αC and βC domains function as a structural “scaffold” of the TCR, helping to maintain the overall stability of the molecule during protein folding and assembly with the CD3 complex [3]. Insufficient stability of the constant domains can lead to protein misfolding, trigger ER stress, and increase degradation through intracellular quality control mechanisms [19]. This factor is particularly important in the context of exogenous TCR expression, such as in TCR-engineered T cell therapies, where TCRs are introduced into T cells via viral vectors [22]. Moreover, high-affinity TCRs or TCRs carrying artificial mutations designed to enhance antigen recognition are often associated with an increased risk of reduced constant domain stability, which in turn negatively affects surface expression levels and the biological function of the TCR [23]. The structural stability of the constant domains highlights the critical role of disulfide bond formation in this context [23]. The conserved disulfide bridge between the αC and βC domains plays a crucial role in maintaining the correct three-dimensional structure of the TCR, thereby stabilising the αβTCR heterodimer. This covalent linkage enhances the receptor’s resistance to conformational destabilisation during biosynthesis and surface expression, supporting proper assembly with the CD3 complex [24]. Consequently, intrachain disulfide bonds within the constant domains are essential for ensuring TCR stability, efficient intracellular trafficking, and sustained surface expression [3].

## 4. Interchain Disulfide Bonds in TCR Stabilisation

Disulfide bonds (S–S) are critical structural elements that determine the stability and function of many membrane and secreted proteins, including the TCR [25]. In the context of molecular biology and TCR engineering, disulfide bonds not only preserve the native architecture of the TCR but are also strategically exploited as a tool to enhance the stability and safety of exogenously expressed TCRs [3,26]. Disulfide bonds are covalent S–S linkages formed between two cysteine residues through oxidation of their thiol (–SH) groups [27]. This process predominantly occurs in the oxidising environment of the ER, where catalytic enzymes such as protein disulfide isomerase (PDI) are present [28]. The disulfide bonds stabilise the tertiary and quaternary structures of proteins by limiting the excessive conformational flexibility of polypeptide chains [27]. Formation of S–S bridges reduces the entropy of the unfolded state, thereby improving the efficiency and fidelity of protein folding and enhancing the thermal and conformational stability of the mature protein [28].

**Figure 2:**
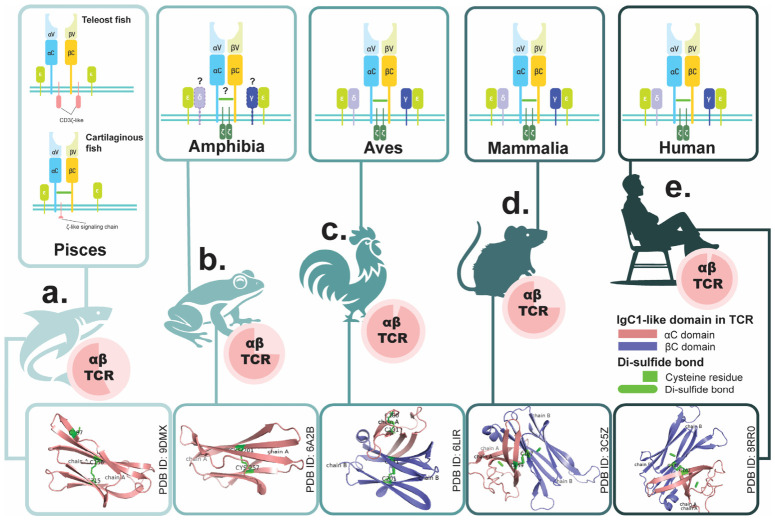
Evolution of the TCR–CD3 system. (**a**) In Pisces, the TCR system (over 60% αβTCR) and CD3 are incomplete, with CD3-like regions and interchain disulfide bonds observed in cartilaginous fish. (**b**) In Amphibia, TCR (over 70% αβTCR) and CD3 gradually mature, though features such as CD3δ, CD3γ, and interchain disulfide bonds remain ambiguous. (**c**) In birds, the TCR (over 90% αβTCR) and CD3 reach a fully formed structure. (**d**) In mammals, such as mice, the TCR system (comprising over 70% αβTCR) is fully established. (**e**) In humans, TCR (over 90% αβTCR) is characterised by αTCR and βTCR chains linked via a single interchain disulfide bond, surrounded by γεCD3, δεCD3, and ζζCD3 complexes.

In native αβTCR molecules, each chain contains intrachain disulfide bonds that help maintain the characteristic immunoglobulin-like domain structure [3]. The emergence of the interchain disulfide bond is closely associated with the evolution of organismal complexity and the progressive structural refinement of the TCR complex (Figure 2). Additionally, an interchain disulfide bond covalently links the αTCR and βTCR chains; however, this bond provides limited mechanical stability, particularly under conditions where multiple distinct TCRs are co-expressed within the same cell [23,29]. Concurrent presence of endogenous and transgene-encoded TCRs increases the risk of mispairing between non-cognate αTCR and βTCR chains [30]. Such mispairing not only reduces the surface density of the desired TCR but also poses the risk of generating hybrid TCRs with unpredictable antigen specificities.

Following translation on ER-bound ribosomes and translocation into the ER lumen via the Sec61 translocon, each αTCR and βTCR chain first folds independently, forming the intradomain disulfide bonds characteristic of Ig-like domains. Once the variable and constant domains reach an appropriate three-dimensional conformation, αTCR and βTCR subsequently associate through noncovalent interactions, thereby bringing two conserved cysteine residues located in the constant regions, αC and βC, into close proximity. Catalysed by members of the protein disulfide isomerase family, particularly PDI and ERp57, the two thiol (–SH) groups are oxidised to form an interchain disulfide bond, which structurally stabilises the αβTCR heterodimer. Only correctly folded αβTCR molecules containing a fully formed interchain disulfide bond can pass ER quality-control mechanisms, allowing assembly with the CD3 complex and subsequent transport to the cell surface, where the receptor mediates peptide–MHC recognition and initiates immune signalling [28].

The formation of a single interchain disulfide bond is highly dependent on the spatial geometry of the αTCR and βTCR chains and is therefore non-random [28]. In humans, the constant regions of both the α and β chains adopt an IgC1-like fold, forming a β-sandwich composed of 7–9 β-strands and creating a relatively flat α–β interface [23,29,31]. The natural occurrence of additional interchain disulfide bonds (e.g., arising from mutation) may introduce steric clashes, distort the native fold, and impair CD3 association as well as membrane stability of the TCR complex [16,29]. Under physiological conditions, interaction between the two chains in this region involves only a single cysteine pair positioned at an optimal distance of approximately 2.03–2.05 Å, with side-chain orientations compatible with disulfide bond formation [3].

## 5. A Second Interchain Disulfide Bond Links the TCR Constant Domains

Thus far, some studies have described the formation or deliberate engineering of a second disulfide bond between αTCR and βTCR to enhance heterodimer stability (Table 1) [16,29,32,33]. This additional disulfide bond is typically introduced by the rational insertion of novel cysteine residues at selected positions within the constant regions or at the junction between the variable and constant domains of the two chains [34]. During biosynthesis in the ER, these engineered cysteines—analogous to the native interchain disulfide bond— can undergo oxidative coupling mediated by the ER’s PDI, resulting in the formation of a second interchain disulfide bond [27,35]. The presence of this additional bond increases the structural stability of the αβTCR complex, reduces chain dissociation or mispairing with endogenous TCR chains, and improves assembly with the CD3 complex as well as surface expression [29]. Consequently, the second interchain disulfide bond not only reinforces the spatial integrity of the TCR but also has important functional implications in TCR engineering strategies, particularly in TCR-T cell therapy, by enhancing receptor stability and signalling sensitivity. Introduction of a second interchain disulfide bond has been shown to markedly improve these parameters: in primary human T cells, cysteine-modified TCRs achieved ~2-fold higher surface expression (70% vs. 35% tetramer-positive cells; MFI 151 vs. 85, *p* < 0.001 across 13 experiments with 10 donors) and superior antitumor activity compared with wild-type versions [29].

**Figure 3:**
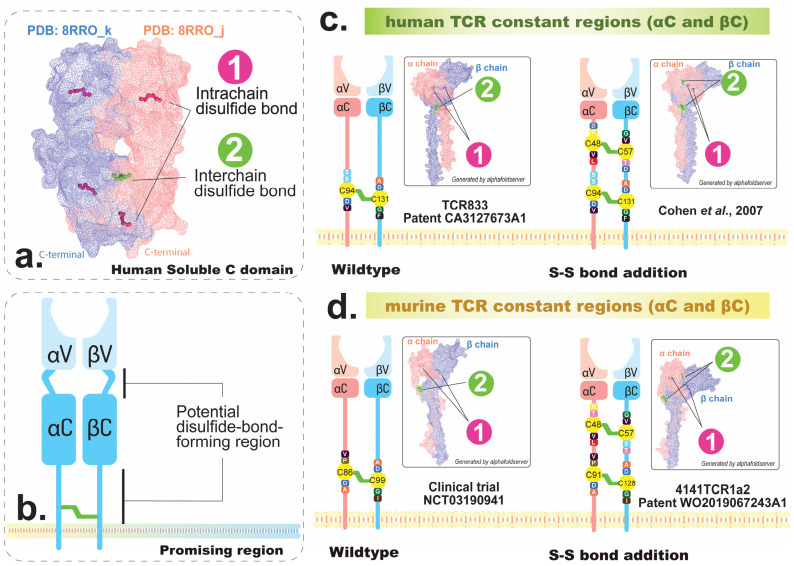
TCR structures with a second interchain disulfide bond. (**a**) Crystal structure of the constant region (without transmembrane domain) of a TCR recognising the Kras G12V neoantigen, showing the canonical four intrachain disulfide bonds and one interchain disulfide near the C-terminal of the peptide chain. (**b**) Structural regions with potential for residue modification to introduce an additional interchain disulfide bond. (**c**) TCR with human constant region: wild-type and engineered with a second interchain disulfide [29]. (**d**) TCR with mouse constant region: wild-type and engineered with a second interchain disulfide.

The second bond has been shown to induce structurally significant changes in the T-cell receptor (Figure 3). Directed mutagenesis studies combined with crystallographic structural analyses have demonstrated that increasing the number of covalent linkages between the α and β chains enhances thermal stability and reduces the tendency of the heterodimer to dissociate, particularly under mechanical stress during interactions with pMHC complexes [32,36]. Molecular dynamics simulations and comparative structural analyses between native TCRs and variants reinforced by additional disulfide bonds further predict that the relative flexibility between the variable and constant domains is restricted, leading to a geometric “locking” of the overall TCR framework [37,38]. Moreover, High-resolution crystal structures of engineered TCRs confirm that the introduced Cysteines relax into the ideal canonical geometry (S–S distance ~ 2.05 Å, αC–βC distance 3.8–4.5 Å) without inducing steric clashes [36,39].

Although the second bond does not lie directly within the CDRs, high-resolution structural studies have shown that stabilisation of the framework domains indirectly influences the orientation and conformational uniformity of the CDR loops, thereby possibly reducing conformational heterogeneity at the pMHC-binding interface [40,41]. In parallel, cryo-EM data of the TCR–CD3 complex indicate that the precise geometry of the TCR constant regions is a critical determinant for efficient assembly with CD3 chains, and reinforcement of the αC–βC interface by an additional disulfide bond helps maintain an optimal conformation for TCR–CD3 interactions, thereby improving the stability of the entire signalling complex at the cell membrane [17,29,32].

Single-molecule force spectroscopy experiments and mechanical modelling have reinforced the view that the TCR functions as a mechanosensor, in which the stiffness of the αβ axis directly affects the efficiency of force transmission from pMHC to CD3 [17,42]. Enhancing interchain disulfide bonding has been proposed to modify these mechanical properties in a manner that increases mechanotransduction efficiency, thereby augmenting TCR signalling sensitivity [26,43,44].

These structural and functional insights have been directly exploited in TCR engineering, as TCRs designed to incorporate a second interchain disulfide bond exhibit reduced mispairing with endogenous TCRs, increased surface expression, and improved long-term stability, while maintaining or enhancing antigen recognition in preclinical TCR-T therapy models [32,45]. Taken together, experimental and structural evidence indicate that the second disulfide bond not only mechanically reinforces the αβTCR heterodimer but also reshapes its structural dynamics in a manner favorable for CD3 assembly and efficient immune signal transduction.

**Table 1:** Quantitative Improvements from Introduction of a Second Interchain Disulfide Bond in αβTCR Engineering.

Study (Year)	TCR Specificity/Model	Surface Expression Improvement	Mispairing/Pairing Effect	Functional Outcome	Key Notes/References
Cohen et al. (2007) [29]	MART-1 (F4 & F5) & p53-specific; human PBLs (mRNA electroporation)	~2-fold increase (e.g., F4: 70% vs. 35% tetramer+ cells; MFI 151 vs. 85; *p* < 0.001 across 13 experiments, 10 donors)	Preferential pairing of modified chains; reduced competition with endogenous TCR	Significantly higher IFN-γ & GM-CSF secretion (*p* < 0.001); enhanced specific tumor lysis at multiple E:T ratios; better activity at limiting TCR doses	Cornerstone study; benefits seen in both CD8+ and CD4+ T cells; also effective for partially humanized murine TCRs
Voss et al. (2008) [23]	Multiple human TCRs; primary T cells	Increased surface density of introduced TCR	Markedly reduced mispairing with endogenous chains; favored specific αβ pairing	Improved overall TCR–CD3 assembly and functional avidity	Focused on Cα–Cβ interface design; synergistic with other constant-region modifications
Kuball et al. (2006) [32]	Various human TCRs introduced into T cells	Enhanced matched pairing and surface expression	Reduced formation of mismatched hybrids	Improved functional expression and signaling	Early demonstration that a single additional disulfide bond facilitates proper chain pairing
Thomas et al. (2019) & related framework studies [46]	Multiple human TCRs (weak vs. dominant)	2–6-fold increase when combined with framework changes (disulfide often used in platform)	Further reduction in mispairing when paired with murinization or framework engineering	Higher antigen-specific responses	Shows second disulfide as complementary to other strategies (e.g., murine constant regions)
Recent platforms (2020–2025, e.g., Shafer et al., clinical-stage TCR-T) [47]	Neoantigen-specific TCRs in modern vectors ± CRISPR KO	Consistent ~1.5–2.5-fold higher expression; near-complete pairing when combined with KO	Minimal residual mispairing (<5% in optimized systems)	Enhanced persistence, cytokine production, and antitumor efficacy in preclinical models	Routinely incorporated in current TCR-T pipelines; often combined with murinization or endogenous TCR knockout

## 6. Influence of an Additional Interchain Disulfide Bond on the Folding of TCR

The autonomous folding of αTCR and βTCR proceeds in a tightly regulated sequence within the ER, beginning with the formation of intradomain disulfide bonds characteristic of the immunoglobulin superfamily, followed by non-covalent pairing of the two chains, and culminating in covalent stabilization of the heterodimer [28]. The introduction of a second interchain disulfide bond exerts both direct and indirect effects on these steps, particularly at the level of TCR folding intermediates. At the intrachain level, mutagenesis and biochemical analyses indicate that the additional disulfide bond does not disrupt the formation of native intradomain disulfide bonds within the αV, βV, αC, and βC domains, provided that the engineered cysteines do not spatially or kinetically compete with native intradomain cysteine pairs [23,29,48]. Non-reducing SDS–PAGE and pulse–chase experiments have shown that TCRs harboring a second disulfide bond still form fully correct Ig-like domains, indicating that the fundamental folding of each individual chain is preserved [23].

In the interchain folding process, the second disulfide bond is considered to alter the folding energy landscape of the αβTCR heterodimer [49]. Computational simulations and folding kinetics analyses suggest that the early emergence of an additional covalent linkage between the two chains stabilizes productive on-pathway intermediates while reducing the accumulation of misfolded or partially folded off-pathway intermediates [48]. This effect is reflected in a reduced residence time of incompletely assembled TCR species within the ER and a more rapid progression toward the fully folded heterodimeric state. From a cell-biological perspective, these effects would be expected to reduce dependence on ER chaperones [48]. Although not directly measured, the enhanced folding and assembly kinetics of TCRs containing a second interchain disulfide bond are consistent with reduced interactions with ER chaperones, such as BiP and calnexin/calreticulin [23,48]. In parallel, improved folding efficiency would be expected to reduce the fraction of molecules routed to ER-associated degradation. Non-reducing SDS-PAGE and pulse-chase experiments confirmed correct folding, while surface expression assays revealed significantly elevated levels of fully assembled TCR–CD3 complexes (up to 2-fold higher mean fluorescence intensity), reflecting more efficient escape from ER quality control and reduced mispairing [23,29]. The robust surface expression of functional TCRs lowers the activation threshold against low-density antigens and enhances functional avidity, while concurrently minimizing the risk of mispairing-induced off-target toxicity [46,50]. Another important consequence of the second disulfide bond is the reduction in folding competition and mispairing in systems that co-express multiple TCR chains, such as in TCR engineering or TCR-T settings [51]. Early “locking” of the αTCR–βTCR interaction by an additional linkage increases the specificity of chain pairing, thereby limiting the formation of undesired heterodimers or unstable hybrid structures, which otherwise can impose a substantial folding burden on the ER.

In terms of thermodynamic stability, the formation of an additional covalent bond between the two peptide chains significantly decreases the entropy (∆S) of the unfolded state [39]. Because the protein chain is sterically constrained and cannot fully adopt a random coil configuration, the Gibbs free energy (∆G) required for denaturation increases, thereby elevating the melting temperature (Tm) of the TCR by 3 °C to 8 °C and effectively preventing protein aggregation [52,53]. Additionally, regarding the kinetics of complex binding with the peptide-MHC (pMHC), the framework structure—pre-organized and locked by this disulfide bond—reduces the conformational energy barrier upon antigen engagement. Consequently, the association rate constant (k*_on_*) remains stable or increases slightly, whereas the dissociation rate constant (k*_off_*) decreases sharply due to the enhanced stability and prolonged half-life of the TCR-pMHC complex [29,54]. This simultaneous optimization of both kinetic rate constants results in a drastic drop in the equilibrium dissociation constant (K_D_ = k*_off_*/k*_on_*), demonstrating a ten- to thousand-fold increase in thermodynamic binding affinity [55]. Notably, in TCR-T cell therapy applications, this engineered disulfide bond formation possesses a highly favourable free energy effect (a more negative (∆G), which drives the correct pairing between the exogenous chains and suppresses mispairing with endogenous T-cell chains [29,56]. This has particular relevance for TCR-based therapies, where mispairing represents a major risk factor leading to reduced overall therapeutic efficacy [57]. Additionally, there is currently no direct empirical evidence showing that the introduction of a second disulfide bond in the TCR constant region induces T-cell exhaustion. However, this structural modification could theoretically predispose T cells to an exhaustion phenotype through indirect mechanisms. Taken together, this provides a mechanistic foundation for the efficient folding of the TCR and the sustained surface expression of the structure.

## 7. Modulation of TCR–CD3 Assembly by a Second Interchain Disulfide Bond

The presence of a second interchain disulfide bond between αTCR and βTCR induces substantial changes in the assembly of the TCR–CD3 complex. It can begin at the earliest stages of biosynthesis in the ER, as mentioned. Under physiological conditions, only correctly folded, stable αβTCR heterodimers that have achieved an appropriate three-dimensional conformation can pass ER quality-control checkpoints and proceed to assemble with CD3 chains [28]. Heterologous expression systems and directed mutagenesis studies have demonstrated that reinforcing the αβTCR with an additional interchain disulfide bond promotes more efficient formation of stable heterodimers during ER biosynthesis [32,48]. The second bond contributes to stabilizing the geometry of the constant region (αC–βC), which constitutes the direct interface with the εδCD3, εγCD3, and ζζCD3 chains [17,32,48]. High-resolution cryo-EM structures of the TCR–CD3 complex have demonstrated that successful assembly requires high geometric precision, particularly in the relative orientation of the transmembrane segments and the extracellular constant regions of the TCR [17]. By increasing rigidity and reducing conformational fluctuations of the αC–βC framework, the additional disulfide bond can help maintain an optimal configuration for TCR–CD3 interactions, so it can increase the likelihood of successful assembly of the fully competent complex.

Functional evidence for this effect is provided by surface expression assays and flow cytometric analyses, in which TCRs engineered to contain a second interchain disulfide bond display significantly increased surface expression, accompanied by enhanced co-expression of CD3 chains [23,29]. This pattern reflects more efficient TCR–CD3 assembly rather than intracellular accumulation of incompletely assembled αβTCR species within the ER. In parallel, early stabilisation of the αβ heterodimer reduces mispairing between exogenous and endogenous TCR chains, a critical issue in the context of TCR engineering and TCR-T cell therapy [16,23,33]. Furthermore, more efficient assembly of the TCR–CD3 complex in the presence of a second interchain disulfide bond contributes to enhanced stability of the complex at the cell surface [23,29]. These findings are consistent with enhanced surface persistence of TCRs containing a second interchain disulfide bond, suggesting that structural stability established during ER assembly may be retained throughout the receptor’s lifecycle [45]. It should be noted that the influence of the interchain disulfide bond on TCR–CD3 interaction is indirect, operating through stabilisation of the αβTCR heterodimer rather than through direct participation in TCR–CD3 binding [58].

## 8. Strategies for Engineering a Second Interchain Disulfide Bond in TCR

A widely used strategy for introducing a second interchain disulfide bond in the TCR involves the directed introduction of additional cysteine residues into both the αTCR and βTCR chains through amino acid substitution [29,33]. These cysteine residues are selected to be spatially proximal in the three-dimensional structure of the heterodimer, most commonly within the constant domains or at the interface between the variable and constant domains [29,59]. During translation and folding of the TCR, the engineered cysteine residues can spontaneously form a disulfide bond, catalysed by PDI [28]. This approach has been shown to enhance the stability of the αβTCR heterodimer, improve its assembly efficiency with the CD3 complex, and reduce mispairing with endogenous TCR chains, while preserving the ability to recognise peptide–MHC complexes, as mentioned [16,23,29].

The selected positions typically do not correspond to evolutionarily conserved residues, but instead map to framework regions that tolerate cysteine substitution and exhibit favourable spatial geometry for disulfide bond formation [26,32]. The framework regions of the αC and βC constant domains are most frequently targeted, as these regions support stable αβTCR association while remaining distant from antigen-binding CDRs and CD3 interaction surfaces [45,60,61]. Introducing additional cysteines at non-highly conserved positions in these regions enables the formation of a second disulfide bond without disrupting the Ig-like intradomain architecture, while substantially reinforcing the global stability of the αβTCR heterodimer [29]. The success of this strategy is further enhanced by structure-guided disulfide engineering. The structural information derived from X-ray crystallography, cryo-electron microscopy, homology/AlphaFold models of the TCR, or disulfide-substituted TCR (DSS-TCR) is used to identify residue pairs on αTCR and βTCR that exhibit favourable distances and geometries for disulfide bond formation [62,63]. The integration of static structural analysis with molecular dynamics simulations enables the prediction of how the additional disulfide bond will affect the flexibility and rigidity of the αβ axis, allowing for the optimisation of mutation sites to achieve maximal stabilisation without perturbing the CDR regions or intradomain folding [45,60,61].

In addition to the constant domains, the interface between the variable and constant domains (the V–C interface), particularly framework region 4 (FR4) of the αV and βV domains, has also been identified as a suitable site for residue modification [61]. This region functions as a flexible hinge between domains, allowing a certain degree of interdomain mobility in the native TCR [64]. Formation of a disulfide bond at FR4 is proposed to restrict interdomain fluctuations and effectively “lock” the αβ axis into a more stable conformation, while preserving the geometry of the CDRs and the ability to recognise peptide–MHC complexes [23,61,63].

Framework regions of the variable domains—particularly FR2 or FR3 located on the face opposite the CDR loops—may also be exploited to introduce a second disulfide bond, provided that such modifications do not perturb CDR geometry or pMHC recognition [61,63]. These regions do not directly contribute to paratope formation and exhibit relatively low structural variability, thereby allowing substitution of residues such as serine, alanine, or valine with cysteine without grossly impairing the intrinsic folding of the variable domains [61,63,65]. However, selection of such positions typically requires guidance from high-resolution structural data and, in some cases, molecular dynamics simulations, in order to minimise the risk of unintended alterations in CDR orientation [65,66,67]. By contrast, the CDR1–CDR3 loops, the native intradomain cysteines of the Ig fold, and surfaces directly involved in CD3 interactions are generally considered unsuitable for the introduction of additional disulfide bonds, due to the high risk of disrupting folding, inducing ER retention, or impairing antigen recognition [67,68]. Accordingly, most reported engineering approaches favour the introduction of a second disulfide bond within framework regions that exhibit sequence variability but maintain conserved three-dimensional geometry, allowing improved structural stability of the TCR while preserving its fundamental biological function.

Beyond sequence design, the protein expression system plays a decisive role in the efficient formation of the additional disulfide bond. TCRs carrying engineered cysteine residues are typically expressed in eukaryotic systems, particularly mammalian cells, where the ER provides an oxidative environment enriched in enzymes that catalyse disulfide bond formation [28]. Compared with bacterial expression systems, eukaryotic expression allows the second disulfide bond to form in a physiological and coordinated manner during TCR folding and TCR–CD3 assembly, while minimising the accumulation of reduced or misfolded protein species [49,69]. The introduction of a second disulfide bond is invariably accompanied by biochemical and structural validation strategies to confirm the presence and correctness of the engineered linkage [33,70]. Techniques such as non-reducing SDS–PAGE, Western blotting under both reducing and non-reducing conditions, mass spectrometric analysis, and direct structural observation by X-ray crystallography or cryo-EM can be used to clarify the αβTCR heterodimer structure and disulfide bonds [71].

## 9. Current Research Practices and Associated Challenges

**Figure 4:**
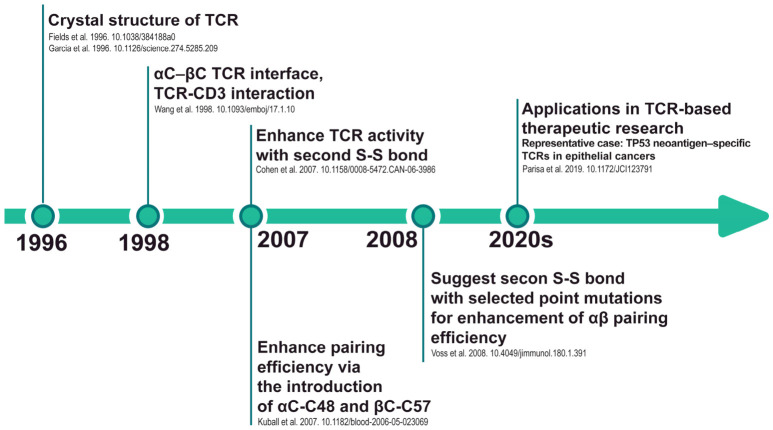
Discovery and development of the second interchain disulfide bond in TCRs, highlighting structural insights and engineering strategies to enhance TCR stability and function [3,23,29,32,33,77,78].

For nearly two decades, the introduction of a second interchain disulfide bond between αTCR and βTCR has emerged as a potential engineering strategy in TCR-T research, aimed at enhancing the heterodimer’s structural stability and pairing fidelity (Figure 4). These improvements are particularly significant in the context of viral vector-mediated TCR expression, where competition between exogenous and endogenous TCR chains represents a major contributor to reduced efficacy and potential safety risks [72]. However, in practice, the implementation of this strategy is not universal and often requires case-by-case optimisation for individual TCRs [73]. One of the primary challenges lies in selecting appropriate cysteine positions that permit disulfide bond formation without perturbing the intrinsic folding of the Ig-like domains or altering the geometry of the CDR regions [61]. Structurally “tolerant” positions are not necessarily compatible with all TCR backbones, particularly in TCRs derived from rare repertoires or those subjected to artificial affinity enhancement. Consequently, in many cases, rational introduction of a second disulfide bond relies on high-resolution structural information and/or molecular dynamics simulations to minimise the risk of misfolding or ER retention [29,74].

A further challenge involves balancing structural stabilisation with the conformational flexibility required for optimal antigen recognition [63]. The additional disulfide bond reinforces the αβTCR heterodimer; excessive rigidification of the interchain axis may restrict the subtle conformational motions required for effective mechanotransduction during TCR engagement with peptide–MHC complexes [23,29]. Accumulating evidence suggests that a defined degree of interdomain flexibility within the TCR contributes to optimal ZAP-70 recruitment and downstream signalling [14,75]. As a result, over-stabilisation of the TCR structure may, in some contexts, lead to diminished functional potency despite increased surface expression.

From a clinical development and manufacturing perspective, the introduction of a second disulfide bond also poses challenges related to product consistency and quality control. Disulfide bond formation is tightly dependent on the oxidative environment of the ER and the activity of catalytic enzymes such as PDI [28]. The process of expanding cells to reach the necessary quantity for therapeutic use is imperative. In large-scale cell manufacturing systems, minor variations in culture conditions can significantly impact the efficiency and fidelity of disulfide bond formation, resulting in structurally heterogeneous TCR populations. This consideration helps explain why, in several clinical programs, disulfide-based stabilisation strategies are often combined with or substituted by alternative approaches such as constant-region murinization or endogenous TCR gene editing. Currently, to overcome challenges in large-scale manufacturing, many bioreactor systems have focused on addressing TCR structural heterogeneity, most notably through Tide Motion^®^ technology. By maintaining a highly stable microenvironment with uniform oxygen and pH levels through direct gas exchange and an agitation-free mechanism, this system ensures precise disulfide bond formation. Consequently, it enhances the structural fidelity and consistency of TCR populations throughout the entire cultivation process [76].

Finally, despite compelling preclinical evidence and patent literature supporting the potential of a second interchain disulfide bond, direct clinical evidence remains limited, largely because detailed residue-level structural designs are rarely disclosed in human clinical trials. This lack of transparency poses challenges for independent assessment of the long-term impact of this strategy on efficacy and safety. In this context, the introduction of an additional disulfide bond is currently regarded as a powerful yet auxiliary engineering tool that should be flexibly integrated with complementary strategies to achieve an optimal balance between structural stability, biological function, and clinical translatability.

## 10. Concluding Remark

Engineering a second interchain disulfide bond in the αβ TCR constant domain has moved from a structural curiosity to a reproducible and mechanistically well-understood strategy for improving TCR-T cell therapy. Across nearly two decades of preclinical work, the approach has consistently delivered three core benefits: reduced mispairing with endogenous TCR chains, higher surface expression of the therapeutic receptor, and enhanced antitumour function—all without fundamentally disrupting antigen recognition.

Yet the field has reached a point where incremental validation is no longer sufficient. Several priorities deserve focused attention. First, the balance between structural rigidity and the conformational flexibility required for mechanotransduction remains incompletely defined; determining the “optimal stiffness window” for different TCR backbones would transform disulfide engineering from an empirical exercise into a rational design process. Second, manufacturing reproducibility must be addressed systematically—variability in disulfide bond formation across large-scale cell expansion remains a genuine barrier to clinical translation, and emerging bioreactor technologies such as Tide Motion^®^ represent a promising but underexplored solution. Third, the field needs prospective clinical data: current evidence is almost entirely preclinical, and the absence of disclosed structural designs in vivo and in clinical trials limits the ability to draw meaningful conclusions about long-term safety and efficacy.

The second disulfide bond is best understood not as a standalone fix but as one layer in a multi-strategy engineering framework, alongside murinization, endogenous TCR knockout, and vector optimisation. Used in this way, it has a credible role in raising the consistency and potency of TCR-T cell products. Realising that potential fully will require closer collaboration between structural biologists, cell engineers, and clinical teams—and a greater willingness to share the molecular details that make independent validation possible.

## Data Availability

Not applicable.

## Abbreviations

The following abbreviations are used in this manuscript:

TCR	T cell receptor
MHC	Major histocompatibility complex
APC	Antigen-presenting cells
CDRs	Complementarity-determining regions
pMHC	Peptide–MHC
ER	Endoplasmic reticulum
